# Transcriptomic analysis reveals that high-protein artificial diet enhances reproductive performance of *Protaetia brevitarsis* (Lewis) (Coleoptera: Scarabaeidae)

**DOI:** 10.1093/jisesa/ieag112

**Published:** 2026-10-05

**Authors:** Ziting Cheng, Qi Peng, Lili Geng, Jie Zhang, Ting Geng, Kui Wang, Changlong Shu

**Affiliations:** State Key Laboratory for Biology of Plant Diseases and Insect Pests, Institute of Plant Protection, Chinese Academy of Agricultural Sciences, Beijing, China; State Key Laboratory for Biology of Plant Diseases and Insect Pests, Institute of Plant Protection, Chinese Academy of Agricultural Sciences, Beijing, China; State Key Laboratory for Biology of Plant Diseases and Insect Pests, Institute of Plant Protection, Chinese Academy of Agricultural Sciences, Beijing, China; State Key Laboratory for Biology of Plant Diseases and Insect Pests, Institute of Plant Protection, Chinese Academy of Agricultural Sciences, Beijing, China; Key Laboratory of Agri-products Quality and Biosafety, Ministry of Education, School of Plant Protection, Anhui Agricultural University, Hefei, China; State Key Laboratory for Biology of Plant Diseases and Insect Pests, Institute of Plant Protection, Chinese Academy of Agricultural Sciences, Beijing, China; Langfang Station for Scientific Monitoring and Experiment on Crop Pests, China MOA (Ministry of Agriculture), Langfang, China; State Key Laboratory for Biology of Plant Diseases and Insect Pests, Institute of Plant Protection, Chinese Academy of Agricultural Sciences, Beijing, China; Key Laboratory of Agri-products Quality and Biosafety, Ministry of Education, School of Plant Protection, Anhui Agricultural University, Hefei, China; State Key Laboratory for Biology of Plant Diseases and Insect Pests, Institute of Plant Protection, Chinese Academy of Agricultural Sciences, Beijing, China; Langfang Station for Scientific Monitoring and Experiment on Crop Pests, China MOA (Ministry of Agriculture), Langfang, China

**Keywords:** artificial feed, transcriptomics, gut differentially expressed gene, nutritional digestion characteristics, reproductive performance

## Abstract

Insects have emerged as promising sustainable alternative protein sources, among which *Protaetia brevitarsis* (Lewis) (Coleoptera: Scarabaeidae) exhibits considerable potential for valorizing lignocellulosic wastes (e.g. crop straw) into high-value proteins. The larvae of this species are traditionally used in medicine and as a health food, but the underdeveloped rearing technology for adults has impeded large‑scale commercialization and application. Thus, the development and optimization of artificial diets represent a critical breakthrough for overcoming this bottleneck. Herein, we integrated expression profile analysis to investigate the molecular mechanisms underlying nutritional digestion in *P. brevitarsis* and the regulatory effects of different diets on its growth and reproductive performance. Transcriptomic analyses further unveiled the core nutritional metabolic traits of *P. brevitarsis*: the larval midgut exhibited broad and high-level expression of genes involved in carbohydrate, fat, and vitamin digestion and absorption, supporting active metabolism of multiple nutrients; in contrast, adults displayed a pronounced demand for proteins. Notably, although adult intestines exhibited fewer total expressed genes, the protein digestion and absorption pathway was exceptionally active, highlighted by 7 core protease genes each displaying a Fragments Per Kilobase of transcript per Million mapped reads value exceeding 10,000. This pattern underscores the high protein demand of adults, laying a robust theoretical foundation for the artificial rearing of *P. brevitarsis* and the precise formulation of artificial diets. Feeding trials demonstrated that adults fed a high-protein artificial diet exhibited significantly increased body weight from the third week post-feeding, with cumulative egg production 1.62-fold higher than that of the apple-fed control group. Furthermore, this study provides valuable insights for the development of artificial diets for economically important insects and the sustainable advancement of the insect rearing industry, ultimately contributing to mitigating global protein demand pressures.

## Introduction

With the rapid global population growth and continuous improvement in living standards, the demand for protein-rich foods has increased significantly (Akhtar and Isman 2018). Traditional livestock and poultry breeding, which relies primarily on soybean meal and fish meal as protein sources, is facing challenges such as limited arable land, environmental pollution, and high production costs. Consequently, there is an urgent need to explore efficient and sustainable alternative protein sources.

Insects have emerged as a highly promising solution due to their high feed conversion efficiency, low environmental impact, and ability to utilize organic waste (van Huis and Gasco 2023). For instance, insects such as *Hermetia illucens* (Linnaeus) (Diptera: Stratiomyidae), *Tenebrio molitor* Linnaeus (Coleoptera: Tenebrionidae), and *Protaetia brevitarsis* (Lewis) (Coleoptera: Scarabaeidae) have been extensively studied for their potential in protein production (Kłobukowski et al. 2025). Among these, *P. brevitarsis* has attracted particular attention due to its unique ability to decompose lignocellulosic biomass, making it a valuable resource for sustainable protein production (Li et al. 2019, Wei et al. 2020). Unlike other insects that require high-quality feed, *P. brevitarsis* can convert lignocellulosic waste (Wang et al. 2022) into high-value proteins (Li et al. 2019, Wei et al. 2020), serving as an industrial species with both economic and ecological benefits. Furthermore, *P. brevitarsis* larvae are rich in essential amino acids, antimicrobial peptides, and bioactive substances, holding potential applications in animal feed, medicine, and functional food industries (Park et al. 2024, 2025, Cha et al. 2025, Kang et al. 2025, Lee et al. 2025, Olawuyi et al. 2025, Sim et al. 2025). Recent reviews have highlighted the sustainability of insect farming (Tanga et al. 2021, Barragán-Fonseca and Gómez 2025) and provided methodological frameworks for artificial diet optimization in Coleoptera (Faria et al. 2024, Nguyen and Hong 2026). However, the immature breeding technology for adult *P. brevitarsis* currently limits the large-scale promotion and application of larvae.

The key to achieving large-scale breeding of *P. brevitarsis* lies in the development and application of artificial feed for the breeding stage. Traditional natural feeds (e.g. apples) suffer from obvious seasonal limitations and unstable nutrient composition. Scientifically formulated artificial feed not only ensures a continuous and stable nutrient supply but also significantly improves insect growth performance and reproductive efficiency. Insect growth, development, and reproduction require a balanced nutrient supply; the deficiency or excess of any nutrient in artificial feed can adversely affect insects. Therefore, research on artificial insect feed faces 2 major bottlenecks: insufficient understanding of the physiological and biochemical mechanisms underlying the nutritional regulation of insect population adaptability, and the lack of reliable physiological or biochemical markers to evaluate the adaptability of specific nutrient sources. Notably, the research paradigm in modern animal nutrition has gradually shifted from traditional “dose-effect” relationship studies to investigations into the mechanisms by which nutrients regulate gene structure and expression. In the context of developing such mechanism-guided diets, studies on other economically important Coleoptera, such as *T. molitor*, have established that dietary protein is a critical determinant of adult growth and reproductive performance (Urrejola et al. 2011, Mahmoud et al. 2025). This underscores the general importance of protein nutrition for adult beetles. However, the specific quantitative requirement, optimal dietary protein level, and most importantly, the molecular digestive adaptations underlying protein utilization in adult *P. brevitarsis* remain largely unknown. Elucidating these mechanisms is essential for moving beyond empirical formulation and achieving precise, efficient feeding strategies for this species. With the rapid development of high-throughput sequencing and bioinformatics technologies, the acquisition and analysis of insect genomic and transcriptomic data have become more accessible, providing a new technical approach for in-depth analysis of nutrient-related gene functions.

The insect intestine is the primary site for nutrient digestion and absorption, as well as detoxification metabolism (Wang et al. 2022). It plays a crucial role in the secretion of digestive enzymes, food digestion, and defense against exogenous toxic substances. This study employed transcriptome sequencing technology to systematically compare differentially expressed genes (DEGs) involved in key physiological processes such as intestinal nutrient metabolism, digestion and absorption, and reproductive development between *P. brevitarsis* larvae and adults fed with a natural feed. Additionally, differences in body weight and reproductive capacity between adults fed with artificial and natural feeds were analyzed. The research findings not only provide a theoretical basis for the optimization and improvement of artificial feed for *P. brevitarsis* but also offer references for the development of artificial feed for other important economic insects. By revealing the molecular regulatory mechanisms of feed efficiency, this study will promote the sustainable development of the insect breeding industry and provide new solutions to address global protein demand challenges. We hypothesized that adults have a higher requirement for dietary protein and would respond positively to a high-protein diet.

## Materials and Methods

### Insect Colony

Adult *P. brevitarsis* were obtained from a laboratory colony maintained at the Langfang Scientific Research and Pilot Base of the Chinese Academy of Agricultural Sciences. Newly emerged adults (within 2 d of eclosion) were used for experiments. Insects were reared in insect‑rearing boxes (370 mm × 255 mm × 150 mm) with 30 ventilation holes (1 cm in diameter) positioned 3 cm below the upper edge. The boxes were covered with light‑transmitting plastic plates to prevent escape. Oviposition substrate was prepared from spent culture medium of *Flammulina velutipes* (golden needle mushroom), which was fully fermented for approximately 15 days until the pile temperature dropped below 35 °C, then mixed with water to adjust humidity to 60%. A plastic grid (mesh side length approximately 2 cm) was placed on the oviposition substrate to facilitate adult grasping and crawling. The rearing environment was maintained at 28 °C, 70% relative humidity, with a photoperiod of 16L:8D and light intensity of approximately 1000 lux. Natural feed consisted of apples (Shandong Yantai Fuji variety), which have been previously reported to be effective for rearing adults (Yang et al. 2014). The high‑protein artificial diet (referred to as HP‑1, Ruimai Dexing [Beijing] Biotechnology Co., Ltd.) was prepared as follows: 200 g brown sugar, 10 g potassium sorbate, 10 g jelly powder, and 50 g yeast powder were mixed with 730 mL distilled water. The mixture was brought to a boil with continuous stirring, then poured into containers and cooled to room temperature to solidify into a jelly‑like feed.

### Sample Preparation and Transcriptome Sequencing

For transcriptome sequencing, adults maintained on the apple diet for 4 wk were dissected, and the fat body adhering to the intestine and other tissues was carefully removed. The intestine was quickly placed in PBS solution on ice, the attached fat body was stripped off, the intestinal contents were squeezed out, and the intestine was then stored in RNA preservation solution (TIANGEN, Beijing, China). RNA extraction and purification were performed using the TRIzol Reagent kit (Life Technologies, Carlsbad, CA, United States). The treatment included 6 biological replicates, with each replicate consisting of intestines from 4 adults (2 females and 2 males; sex ratio 1:1). RNA integrity was assessed using an Agilent 5400 Bioanalyzer. Transcriptome sequencing was conducted by Beijing Sinobiocore Biological Technology Co., Ltd. Additionally, the transcriptome sequencing data for the larval midgut and hindgut of *P. brevitarsis* used in this study were previously generated and submitted by our group under NCBI SRA accession number SRR14128221∼ SRR5039459 (Supplementary Table S1).

### Transcriptome Analysis and Expression Quantification

To profile gene expression in the *P. brevitarsis* gut, RNA-seq reads were processed and aligned to the *P. brevitarsis* reference genome (Wang et al. 2019). Raw sequencing reads were first assessed for quality using FastQC (https://www.bioinformatics.babraham.ac.uk/projects/fastqc/, v0.11.2), then trimmed to remove adapter sequences and low-quality bases with Trimmomatic (v 0.32) (Bolger et al. 2014). The resulting high-quality cleaned reads were aligned to the reference genome using the splice-aware aligner HISAT2 (v2.0.5) (Kim et al. 2019) under default parameters. Following alignment, transcript assembly and abundance estimation were performed using StringTie (v2.2.1). For gene-level quantification, featureCounts (v 1.5.0-p3) (Liao et al. 2014) was used to count reads mapping to genomic features, and the resulting raw count matrix served as the foundation for all downstream differential expression analyses. Furthermore, to facilitate visualization and cross-sample comparison of expression levels, Fragments Per Kilobase of transcript per Million mapped reads (FPKM) values were calculated from the raw counts and gene lengths.

### Differential Expression Gene Analysis

Differential expression analysis was performed using DESeq2 (v1.30.1) (Love et al. 2014) for all pairwise comparisons among the 3 sample groups (adult intestine, larval midgut, and larval hindgut). All samples for this study were sequenced in a single batch to minimize batch effects. The DESeq2 package inherently accounts for technical variation by performing library size normalization and modeling variance across samples. The *P-*values from the initial hypothesis tests were adjusted using the Benjamini-Hochberg method, and the resulting adjusted *P-*values (padj) were used as one of the criteria for identifying DEGs. Concurrently, the fold change (FC) in gene expression levels between the 2 groups were calculated. Genes were considered differentially expressed if they met the thresholds of |log2FC| ≥ 2 and padj < 0.05. Venn diagrams and volcano plots were generated using TBtools-II (Chen et al. 2023) and R (https://www.r-project.org/, v 3.5.0). KEGG (Kyoto Encyclopedia of Genes and Genomes) pathway enrichment analysis of the DEG sets was conducted separately using KOBAS (v2.0) (Xie et al. 2011), with significantly enriched pathways defined by a *P-*value < 0.05.

### Effects of Different Feeds on Adult Body Weight and Fecundity

For each dietary treatment (apple or HP-1 artificial feed), 3 replicate rearing boxes were used. Each box was prepared with a 3-cm thick layer of oviposition substrate and covered with a plastic mesh (∼ 2 cm mesh size) to facilitate adult crawling (Supplementary Fig. S1). Forty pairs of newly emerged adults (40 females and 40 males; sex ratio 1:1) were placed into each box, which was then covered with a transparent plastic plate. Rearing conditions were as described in the “insect colony” subsection. Both diets were provided once daily at approximately 60 g per box (fresh weight). The amount was adjusted according to insect daily consumption to ensure ad libitum access while minimizing waste. For the apple diet, fresh Fuji apples (Shandong Yantai) were peeled and sliced into small pieces before feeding.

Every week for 4 consecutive weeks, all adults from each box were sieved out (sieve mesh size: 1.5 cm × 0.5 cm) to record survival and measure total body weight per box. After weighing, the adults were returned to their original box. Simultaneously, the oviposition substrate (containing eggs) from each box was collected weekly. Its humidity was adjusted to approximately 60% by adding water, and a layer of pearl cotton was placed on top to maintain humidity. Two weeks after collection, the number of second-instar larvae was counted as the weekly reproductive output. This metric integrates both oviposition success and early egg-to-larva survival; preliminary tests confirmed that egg hatchability did not differ significantly between diets (data not shown).

### Determination of Main Nutritional Components in Different Feeds and Insect Bodies

Apples were peeled, sliced, dried, and ground; the HP‑1 feed powder was tested directly. The main indicators (protein, moisture, fat, carbohydrate, and ash) in feeds were determined with reference to the National Food Safety Standards GB‑T 6432‑2018 (protein), GB‑T 6435‑2014 (moisture), GB 5009.6‑2016 (fat), and GB‑T 6438‑2007 (ash). Carbohydrate content was calculated by difference using the following formula:


Carbohydrate (%)=100-(fat% + protein% + moisture% + ash%)

For adults, after 4 wk of feeding, 2 females and 2 males were collected from each dietary treatment group for protein and fat determination. Protein content was determined using the Kjeldahl method (GB‑T 6432‑2018), and fat content was determined using the acid hydrolysis method (GB 5009.6‑2016). All tests were commissioned to Qingdao Standard Testing Co., Ltd.

### Statistics Analysis

All statistical analyses were performed using OriginPro (version 10.2.0.188). Body weight changes over time were analyzed using a 2‑way repeated measures analysis of variance (ANOVA) with Diet (apple vs. HP-1) as the between subjects factor and time (weeks) as the within‑subjects factor. When a significant interaction was detected, post hoc comparisons at each time point were conducted using Bonferroni’s multiple comparisons test. To compare the total reproductive output over the 4‑wk experimental period, the cumulative egg production curve was plotted for each individual rearing box, and the area under the curve (AUC) was calculated. The AUC values from the artificial diet group (*n *= 3) and the apple group (*n *= 3) were compared using an unpaired *t*‑test with Welch’s correction (which does not assume equal variances between groups).

## Results

### Analysis of Transcriptome-Expressed Genes in the Intestines of *P. brevitarsis* Adults and Larvae

In this study, transcriptome sequencing was performed on the adult intestines (6 samples, pag1-6), larval midguts (6 samples, plm1-6), and larval hindguts (3 samples, plh1-3) of *P. brevitarsis*. All adult intestine samples had RNA Integrity Number (RIN) values ≥ 9.3, confirming high-quality RNA suitable for library construction and sequencing (Supplementary Table S2). A total of 613.33 M raw reads (86.60 Gbp) and 608.97 M clean reads (85.40 Gbp) were obtained. The clean data for each sample exceeded 32 M reads, with Q20 and Q30 base percentages above 97% and 92%, respectively, and a G + C content ranging from 37.85% to 45.22%. Principal component analysis based on the gene expression profiles revealed clear separation between the 3 sample groups (adult intestine, larval midgut, larval hindgut) and tight clustering of biological replicates within each group (Supplementary Fig. S2), confirming high data reproducibility and that the major source of transcriptional variation corresponded to the biological factors of developmental stage and gut region. These results indicate that the quality of the *P. brevitarsis* intestinal transcriptome sequencing is high, meeting the requirements for subsequent data analysis (Supplementary Table S1).

Alignment of the transcriptome data to the *P. brevitarsis* genome (Reference No. ASM 414364v1) showed that an average of 27.47 M reads (62.11%) could be mapped to the reference genome (Supplementary Table S3). This moderate mapping rate is likely influenced by the current completeness of the genome annotation. Crucially, from these aligned reads, an average of 22.39 M high-quality reads per sample were unambiguously assigned to annotated genes (Supplementary Table S4), providing a substantial and reliable dataset for gene expression quantification. Among them, the adult intestine had the highest number of mapped reads, with an average of 30.69 M reads (65.53%) mapped to the reference genome; the larval hindgut had the lowest number of mapped reads, with an average of 25.19 M reads (54.37%) mapped. Similarly, the adult intestine had the highest number of mapped reads to annotated genes (average 24.98 M reads), while the larval hindgut had the lowest (average 19.73 M reads). Ultimately, a total of 27,217 expressed genes were annotated in the adult intestinal transcriptome, including 7,336 low-abundance expressed genes (average FPKM: 0 ∼ 1.00), 6,702 medium-abundance expressed genes (average FPKM: 1 ∼ 10), and 13,179 high-abundance expressed genes (average FPKM: >10). In the larval midgut transcriptome, 29,965 expressed genes were annotated, including 5,720 low-abundance genes, 8,834 medium-abundance genes, and 15,411 high-abundance genes. In the larval hindgut transcriptome, 20,457 expressed genes were annotated, including 2,087 low-abundance genes, 5,817 medium-abundance genes, and 12,553 high-abundance genes (Fig. 1A, Supplementary Table S5). Total expression level statistics showed that the larval hindgut had the highest total expression level (34,693,003), followed by the larval midgut (24,365,801), and the adult intestine had the lowest (16,051,706).

**Fig. 1.**
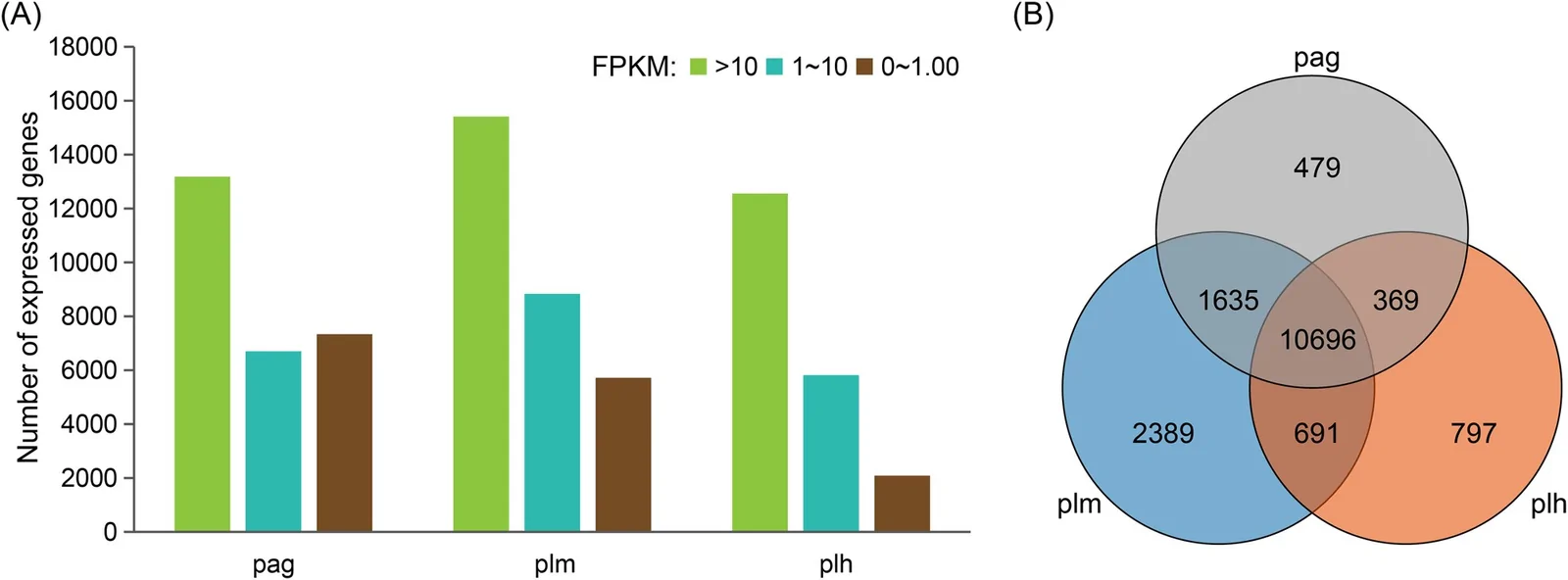
Statistical analysis of expressed genes (A) and distribution analysis of high-expression genes (B) in the intestines of *P. brevitarsis* adults and larvae. pag, adult intestine; plm, larval midgut; plh, larval hindgut.

Analysis of the distribution of high-abundance expressed genes revealed that adults and larvae shared 10,696 high-expression genes. The larval midgut had the highest number of unique high-expression genes (2,389), accounting for 15.50% of all high-expression genes in the larval midgut; the adult intestine had the lowest number of unique high-expression genes (479), accounting for 3.63% of all high-expression genes in adults; the larval hindgut had 797 unique high-expression genes, accounting for 6.35% of its total high-expression genes (Fig. 1B).

To compare the expression changes of important genes in the intestines of adults and larvae, differential expression analysis was performed using DESeq2 software. A total of 11,782 DEGs (padj < 0.05, |log2FC| ≥ 2) showed significant changes in expression levels. Compared with the larval midgut, the adult intestine had 6,079 DEGs, including 1,350 upregulated genes and 4,729 downregulated genes (Fig. 2A). Compared with the larval hindgut, the adult intestine had 5,196 DEGs, including 2,943 upregulated genes and 2,253 downregulated genes (Fig. 2B). Additionally, compared with the larval hindgut, the larval midgut had 8,272 DEGs, including 5,931 upregulated genes and 2,341 downregulated genes (Supplementary Fig. S3). These results indicate significant differences in the intestinal gene expression of *P. brevitarsis* at different developmental stages, suggesting differences in nutritional requirements and digestive absorption characteristics. We note that the lower number of biological replicates for the larval hindgut (*n *= 3) compared to the adult intestine and larval midgut (*n *= 6 each) may reduce the statistical power for detecting DEGs in that specific comparison; however, the substantial number of DEGs still identified underscores a robust transcriptional divergence.

**Fig. 2.**
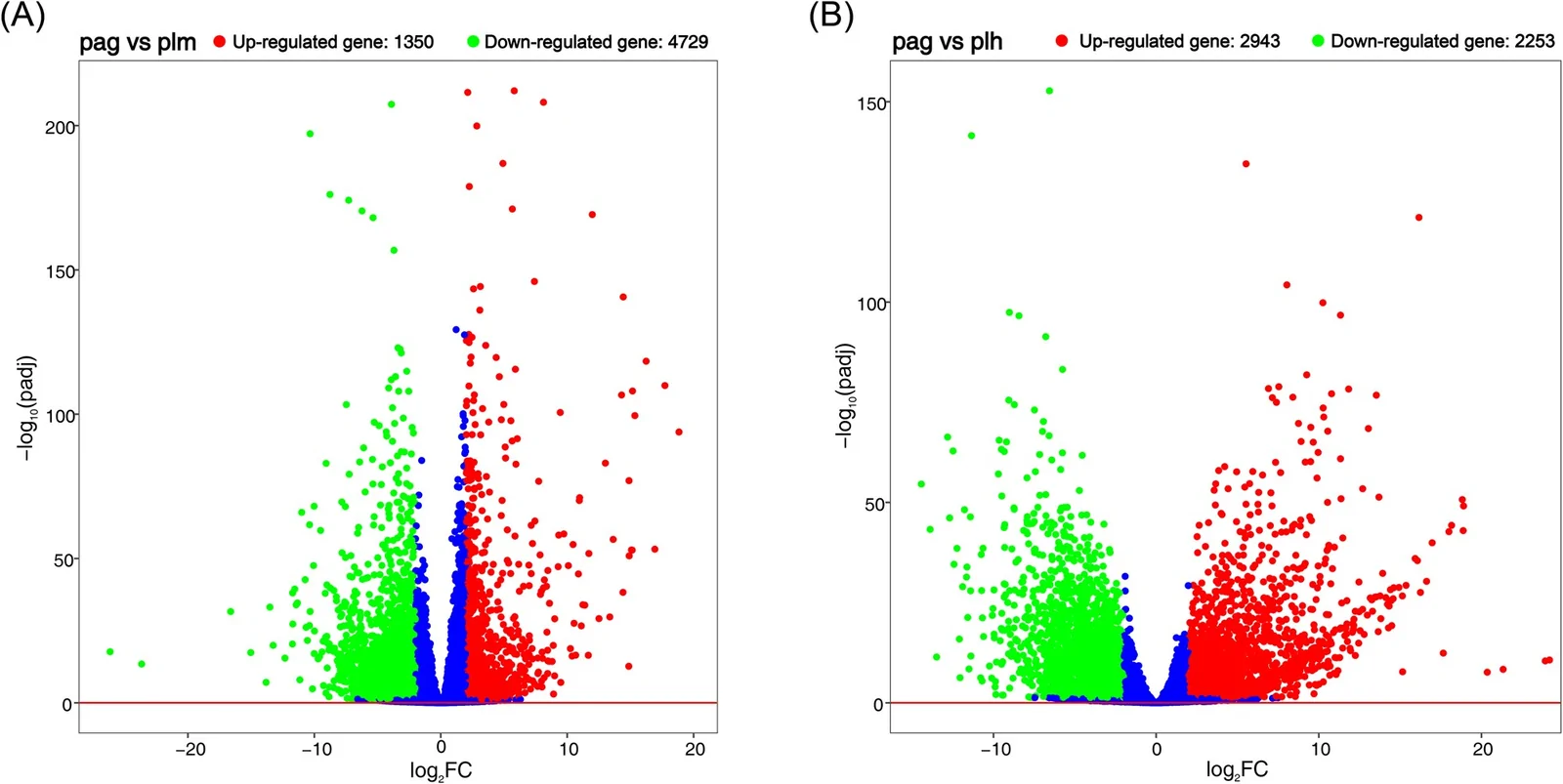
Expression levels of DEGs in the intestines of *P. brevitarsis* adults and larvae. (A) Volcano plot analysis of *P. brevitarsis* adult intestine (pag) vs. larval midgut (plm); (B) volcano plot analysis of *P. brevitarsis* adult intestine (pag) vs. larval hindgut (plh).

### Functional Analysis of Differential Expression Genes

The identified DEGs were aligned to the KEGG database and further mapped to KEGG orthology (KO) for KEGG functional enrichment analysis. The results showed that, compared with the larval midgut, among the 1,350 upregulated genes in the adult intestine, 93 genes were significantly enriched (padj < 0.05) in 6 KO functional terms, namely Lysosome, Pancreatic secretion, Protein digestion and absorption, other glycan degradation, Sphingolipid metabolism, and Antifolate resistance. Among these, the significant enrichment of “Protein digestion and absorption” and “Lysosome” pathways in adults highlights a pronounced shift toward protein utilization at this stage. Except for Antifolate resistance, all other KO functions were related to insect nutrient digestion and absorption. Among the 4,729 downregulated genes, 463 genes were significantly enriched (padj < 0.05) in 40 KO functional terms, including multiple pathways related to insect digestion and nutrient absorption, such as ABC transporters, Lysosome, Glycosyltransferases, Bile secretion, Gastric acid secretion, and Retinol metabolism (Fig. 3).

**Fig. 3.**
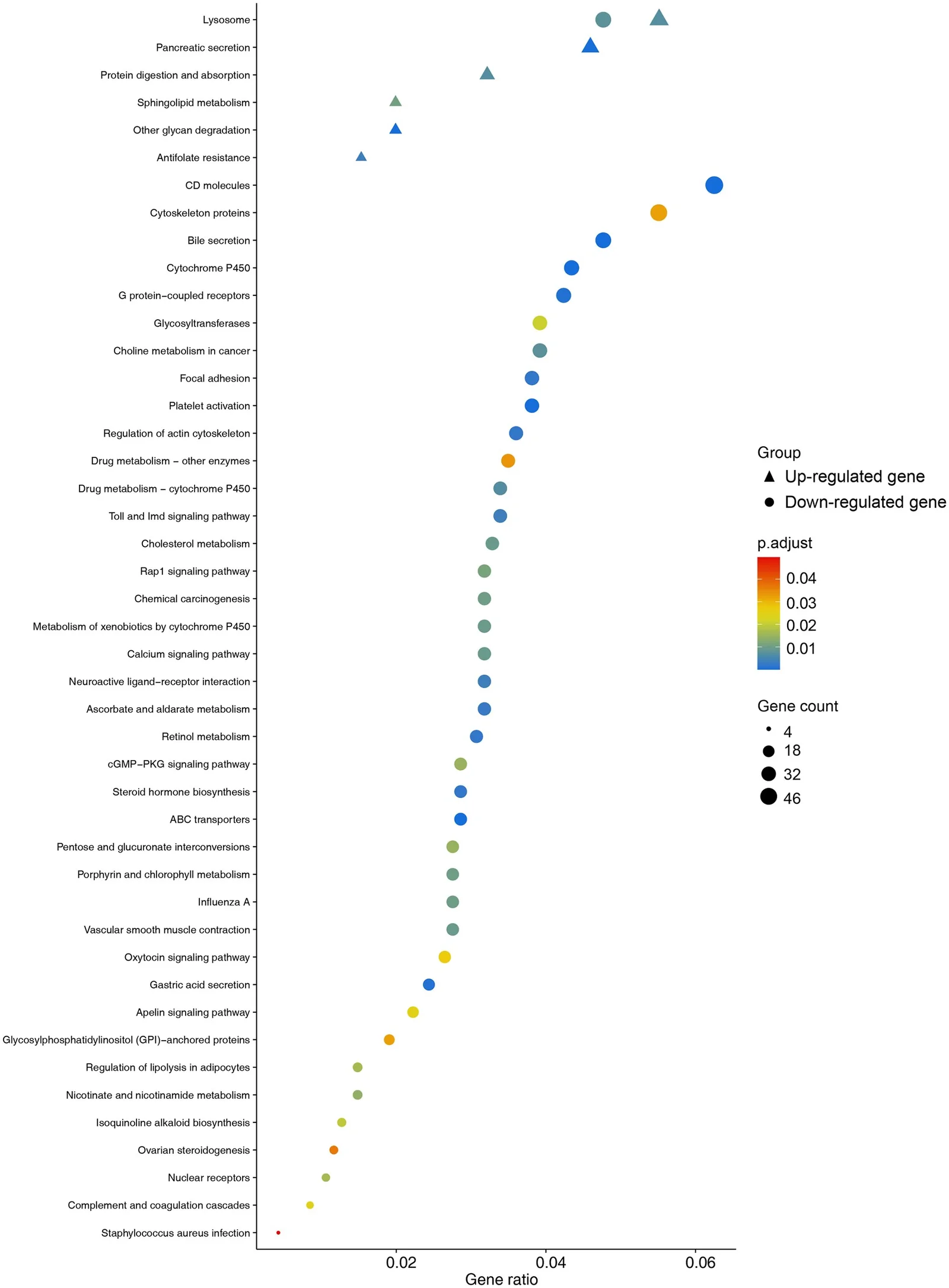
KEGG functional annotation of DEGs between the adult intestine and larval midgut of *P. brevitarsis*.

Compared with the larval hindgut, among the 2,943 upregulated genes in the adult intestine, 553 genes were significantly enriched (padj < 0.05) in 58 KO functional terms. The functions related to insect digestion and nutrient absorption included Protein digestion and absorption, Fat digestion and absorption, Lysosome, ABC transporters, Amino acid-related enzymes, other glycan degradation, and Proteasome. This pattern further corroborates the enhanced capacity of adults for protein and fat processing. Among the 2,253 downregulated genes, 214 genes were significantly enriched (padj < 0.05) in 12 KO functional terms, including multiple pathways related to insect immunity, digestion, and nutrient absorption, such as Cytochrome P450, Toll and Imd signaling pathway, other glycan degradation, glutathione metabolism, tyrosine metabolism, valine, leucine and isoleucine degradation, and glycine, serine and threonine metabolism (Fig. 4).

**Fig. 4.**
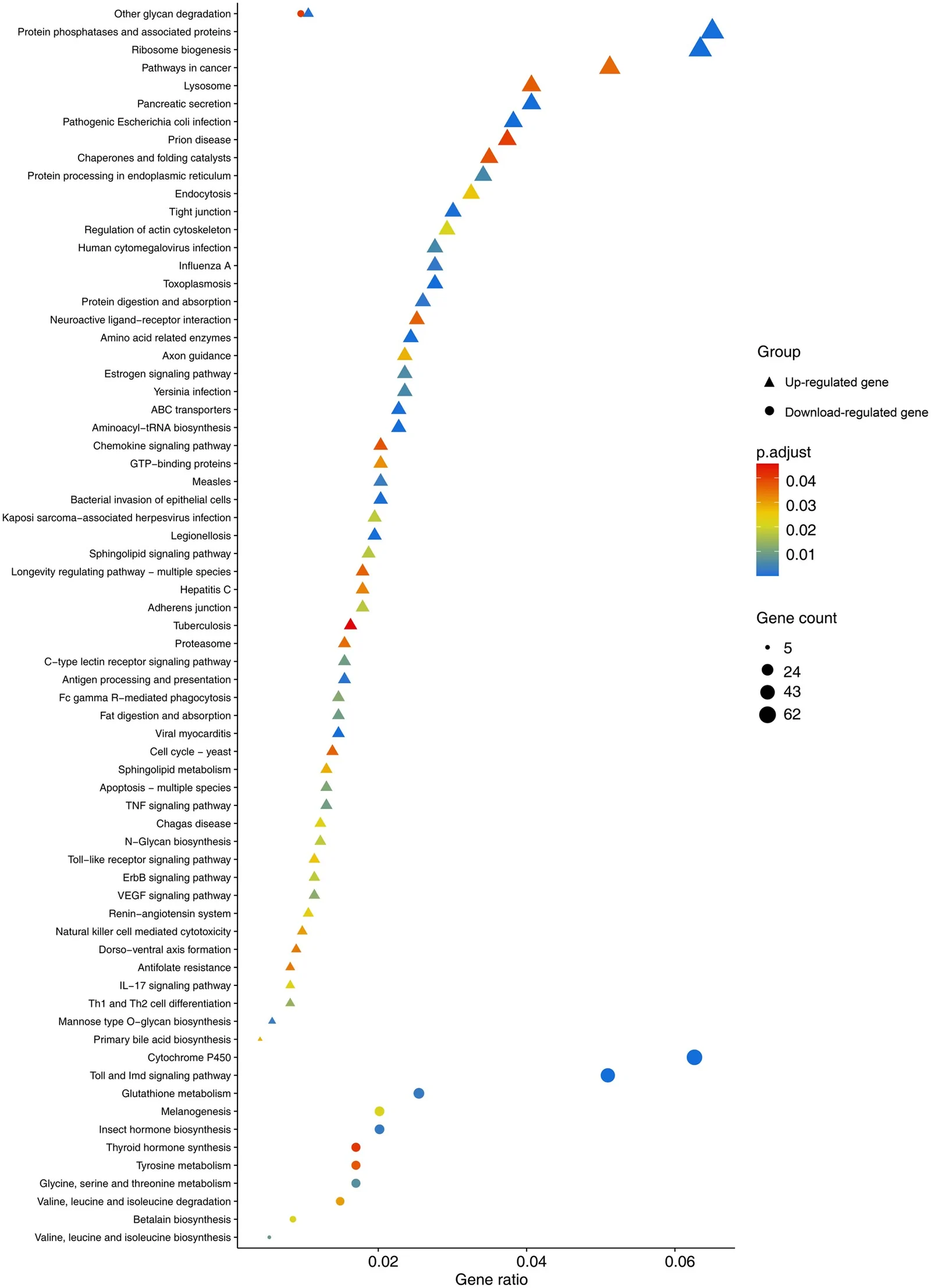
KEGG functional annotation of DEGs between the adult intestine and larval hindgut of *P. brevitarsis*.

In addition, compared with the larval hindgut, among the 5,931 upregulated genes in the larval midgut, 882 genes were significantly enriched (padj < 0.05) in 97 KO functional terms. The functions related to insect digestion and nutrient absorption included protein digestion and absorption, carbohydrate digestion and absorption, fat digestion and absorption, ABC transporters, bile secretion, endocytosis, and pancreatic secretion. The broad enrichment of pathways for carbohydrate, fat, and protein digestion underscores the larval midgut’s role as a central, multifunctional organ for nutrient acquisition from fermented lignocellulose. Among the 2,341 downregulated genes, 319 genes were significantly enriched (padj < 0.05) in 20 KO functional terms, including multiple pathways related to insect digestion and nutrient absorption, such as cytochrome P450, glutathione metabolism, pentose phosphate pathway, other glycan degradation, biosynthesis of unsaturated fatty acids & fatty acid elongation, glyoxylate and dicarboxylate metabolism, glycine, serine and threonine metabolism, one carbon pool by folate, and peroxisome (Supplementary Fig. S4).

### Analysis of Intestinal Nutritional Digestion Mechanisms

Based on the KEGG database, the transcribed genes were mapped to digestion and absorption pathways to systematically analyze the types and quantities of digestion and absorption pathways expressed in the *P. brevitarsis* genome and the intestines of adults and larvae. The main pathways used to predict digestive enzymes and nutrient absorption mechanisms included: carbohydrate digestion and absorption (map04973), protein digestion and absorption (map04974), fat digestion and absorption (map04975), and vitamin digestion and absorption (map04977). The results showed that a total of 290 genes in the *P. brevitarsis* genome were associated with these 4 digestion and absorption pathways (Supplementary Table S6). Tissue-specific expression analysis revealed that 153 highly expressed genes (average FPKM > 10) were detected in the adult intestine, 159 in the larval midgut, and 128 in the larval hindgut (Supplementary Table S7). Overall, the adult intestine and larval midgut exhibited the most diverse digestion and absorption pathways. Further analysis of the common pathway genes among various tissues showed that adults and larvae shared a large number of digestive and absorption pathways, and the number of shared pathways between adults and larval midguts was greater than that between adults and larval hindguts (Fig. 5A). Expression analysis revealed a distinct specialization: the adult intestine displayed the highest activity for the protein digestion and absorption pathway, whereas the larval midgut showed the highest activity for the carbohydrate, fat, and vitamin digestion and absorption pathways (Fig. 5B). The prominence of the protein pathway in adults was driven by the exceptionally high expression of several protease genes (HCG031453, HCG044724, HCG044695, HCG030582, HCG044656, HCG044789, HCG044626), each exceeding 10,000 FPKM, levels which were substantially higher than those in the larval intestine (Supplementary Table S7).

**Fig. 5.**
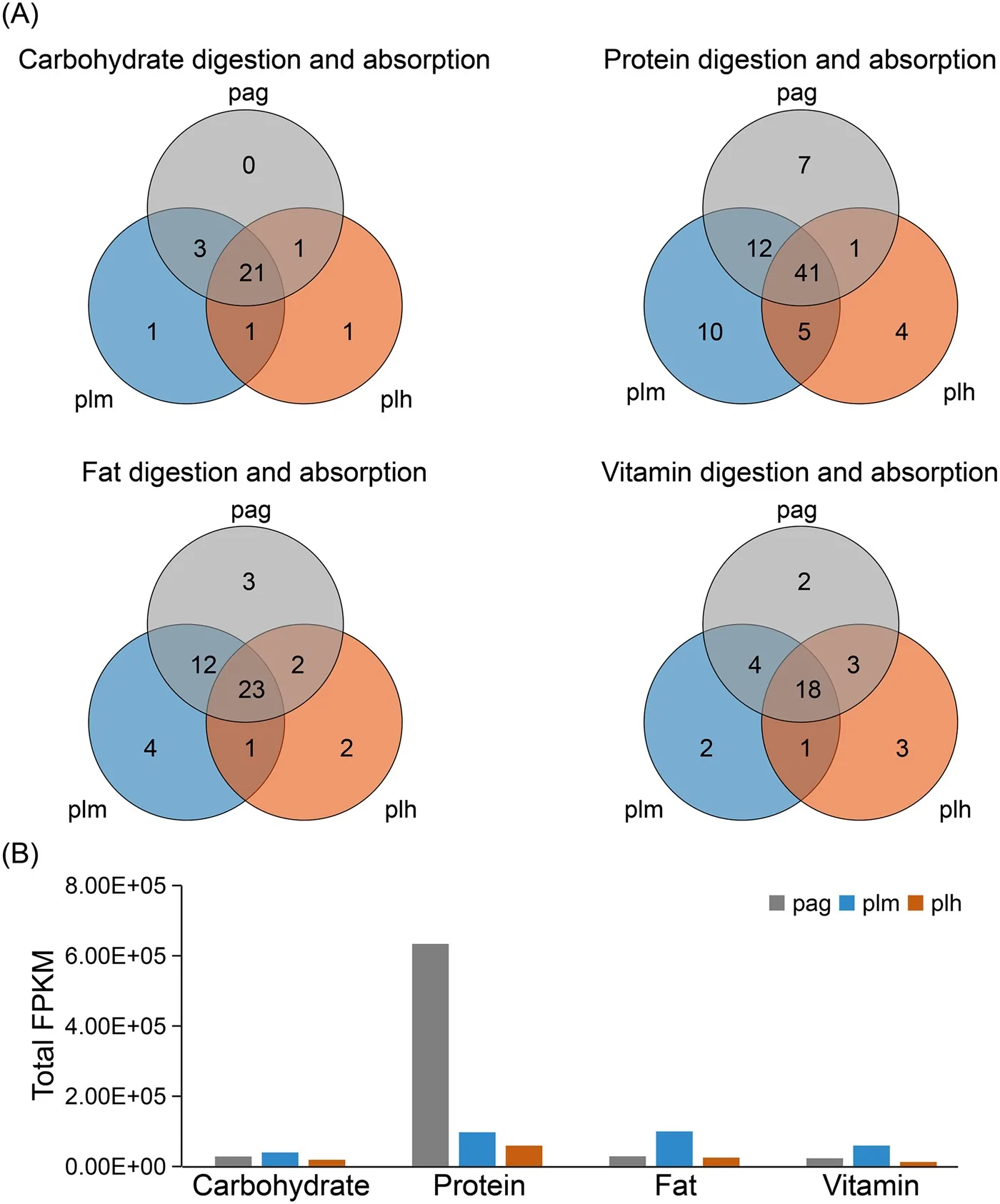
Differential analysis of digestion and absorption pathways in the intestines of *P. brevitarsis* adults and larvae. (A) Comparison of the number of digestion and absorption pathways; (B) comparison of total expression levels. pag, adult intestine; plm, larval midgut; plh, larval hindgut.

### Annotation of Digestive Enzyme Genes in the Genome and Intestinal Transcriptome of *P. brevitarsis*

The aforementioned results indicate that protein-related nutrient absorption is crucial for adults. Based on gene function annotation results, a systematic analysis of the types and quantities of digestive enzyme genes expressed in the *P. brevitarsis* genome and the intestines of adults and larvae was conducted, including trypsin, aminopeptidase, carboxypeptidase, serine protease, metalloprotease, cysteine protease, and aspartic protease.

Genome annotation results showed that a total of 389 digestive enzyme-related genes were identified in the *P. brevitarsis* genome (Supplementary Table S8). Further analysis of the expression of these genes in the intestines of adults and larvae was performed using transcriptome data with a high-expression threshold of FPKM > 10. The results showed that 187 highly expressed digestive enzyme genes were detected in the adult intestine, 204 in the larval midgut, and 174 in the larval hindgut (Supplementary Table S9). Total expression level analysis revealed that the larval midgut had the highest total expression level of digestive enzymes, followed by the adult intestine, and the larval hindgut had the lowest (Fig. 6A). Distribution analysis indicated that adults and the larval midgut shared a large number of digestive enzyme genes (Fig. 6B).

**Fig. 6.**
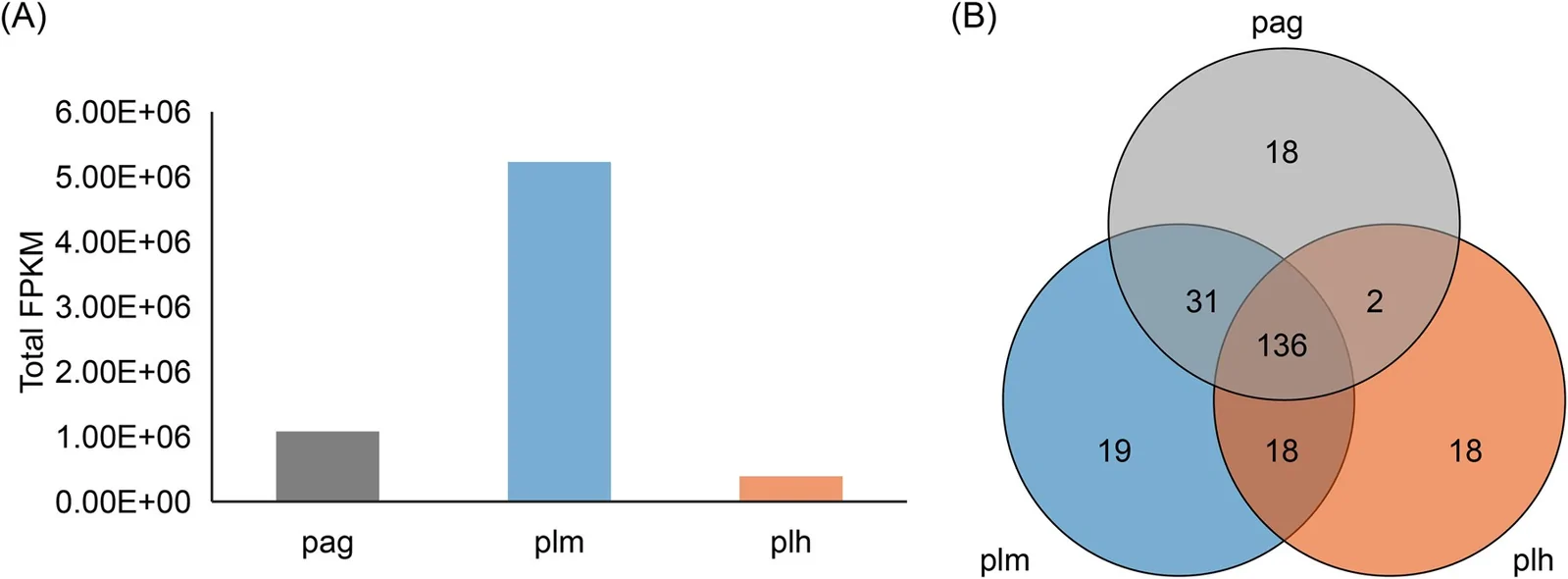
Differential analysis of digestive enzymes in the intestines of *P. brevitarsis* adults and larvae. (A) Total expression level analysis; (B) distribution analysis. pag, adult intestine; plm, larval midgut; plh, larval hindgut.

### Analysis of Nutritional Component Contents in Different Feeds

Transcriptomic analysis results showed that the protein digestion and absorption pathway is the core pathway of intestinal nutrient metabolism in *P. brevitarsis*, and some proteases are specifically highly expressed in the adult intestine, directly reflecting the adults’ high demand for protein nutrition. To further verify the effect of adding protein to the feed, in this study, the artificial feed HP-1 and dried apple pulp (Fuji variety) were first tested to compare the contents of main nutrients such as protein, carbohydrate, and fat (Table 1). The results showed that the protein content of the artificial feed was 13.0%, which was much higher than that of apples (0.92%). Carbohydrates were the main component of both feeds; the carbohydrate content of the artificial feed was 72.8%, while that of apples was higher (87.0%). Fat was the least abundant nutrient in both feeds (less than 1%), with the artificial feed containing 0.8% fat and apples containing 0.9% fat. In addition, the artificial feed had a higher ash content (10.0%) than the natural feed (apples, 4.7%), indicating that the artificial feed was richer in mineral elements.

**Table 1.** Main nutritional component contents of different feeds (g/100 g fresh weight)

Item	Artificial feed HP-1	Natural feed apple
**Protein**	13.0	0.92
**Moisture**	3.43	6.53
**Ash**	10.0	4.7
**Fat**	0.8	0.9
**Carbohydrate**	72.8	87.0

### Effects of Different Feeds on Adult Body Weight

A 2-way repeated measures ANOVA revealed a highly significant interaction between diet and time (*F* = 18.51; df = 4, 16; *P *< 0.0001), indicating that the pattern of body weight change over time differed significantly between the 2 dietary groups (Supplementary Tables S10 and S11). As shown in Fig. 7, the apple‑fed group exhibited rapid initial weight gain, reaching its peak body weight within the first week. Bonferroni’s post hoc tests confirmed that the apple‑fed group was significantly heavier than the artificial feed‑fed group at week 1 (T1, *P *= 0.005; Supplementary Table S12). In contrast, the artificial feed‑fed group gained weight more slowly initially, reaching a level comparable to the apple‑fed group by the second week (T2, *P *= 1.00). However, body weight of the artificial feed‑fed group continued to increase thereafter, becoming significantly higher than that of the apple‑fed group from the third week onward (T3, *P *= 0.001; T4, *P *= 0.001).

**Fig. 7.**
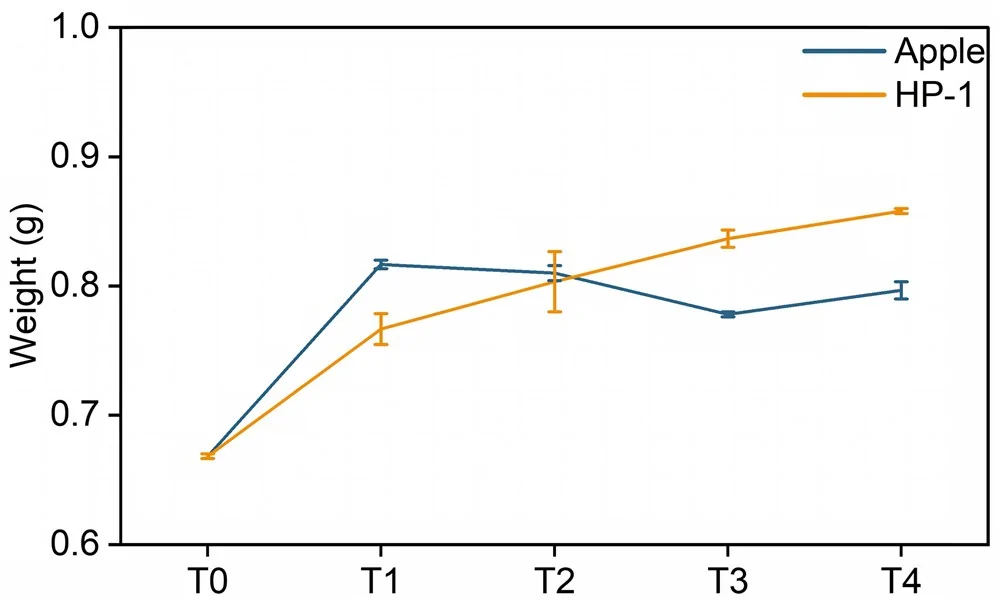
Effects of feeding different feeds on adult body weight. Data points represent mean body weight (± SEM) measured at 5 time points: initial (T0) and at weekly intervals thereafter (T1, T2, T3, T4) (*n *= 3). A 2-way repeated measures ANOVA revealed a significant ‘Time × Diet’ interaction (*F* = 18.51; df = 4, 16; *P *< 0.0001). Significant differences between diets at specific time points, as determined by Bonferroni’s post hoc tests, are indicated: **, *P *< 0.01; ***, *P *< 0.001.

From the body weight gain data, it is possible that insects prefer natural feeds such as apples, leading to rapid and substantial feeding in the early stage to obtain nutrients, thus resulting in faster weight gain. In contrast, the artificial feed provides more abundant nutrients such as proteins, ultimately leading to higher body weights in the artificial feed-fed group.

To verify the above speculation, the protein and fat accumulation in adults fed with different feeds was further determined (Table 2). The results showed that the protein content of adults (both females and males) fed with the artificial feed was higher than that of those fed with apples, being 1.12 times (females) and 1.15 times (males) higher. However, the fat content of adults fed with apples was higher, being 1.76 times (females) and 1.89 times (males) higher than that of the artificial feed-fed group. This may be due to the fact that the fat in apples is derived from living cells and is more easily absorbed. Therefore, the addition of fat-based nutrients should be increased in future feed development.

**Table 2.** Main nutritional component contents of adults (pooled sample) after 4 wk of feeding with different feeds (g/100 g fresh weight)

Item	Apple—Female	Apple—Male	Artificial feed—Female	Artificial feed—Male
**Protein**	24.3	25.7	27.2	29.6
**Fat**	15.0	14.4	8.5	7.6

### Effects of Different Feeds on Adult Egg Production

The total reproductive output over 4 wk, quantified by egg production curve (AUC), was numerically 1.62 times higher in the artificial feed-fed group (mean AUC ± SEM: 49.13 ± 5.20) than in the apple-fed group (30.29 ± 1.21), representing a 62.20% increase (Supplementary Tables S13 and S14). This difference showed a large effect size (*R*² = 0.85). However, in a Welch’s unpaired *t*-test, which does not assume equal variances, this increase did not reach the conventional threshold for statistical significance (*t* = 3.53; df = 2.22; *P *= 0.06). The cumulative egg production curves are shown in Fig. 8.

**Fig. 8.**
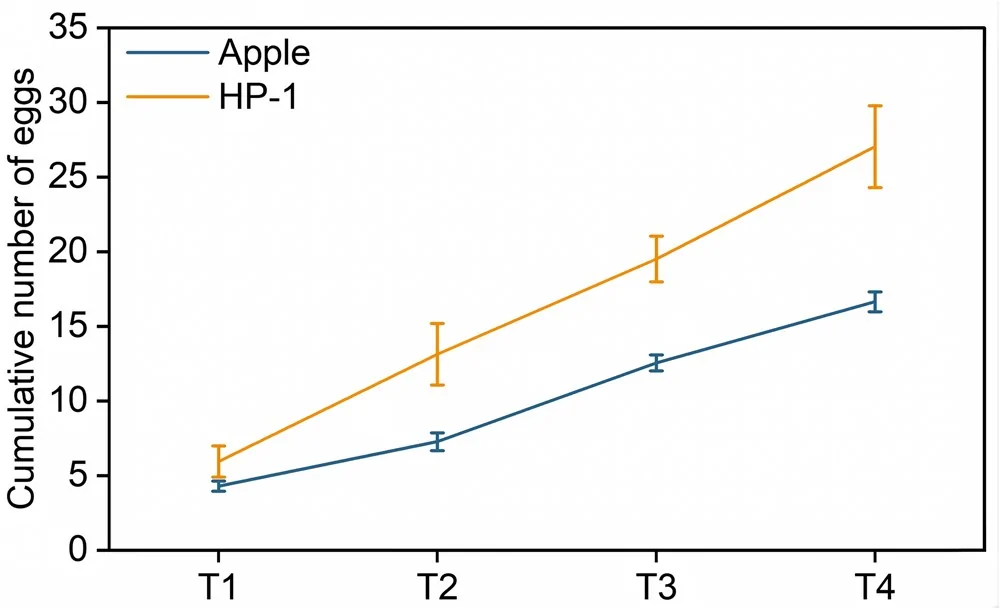
Cumulative egg production data of adults fed with different feeds. Data points represent mean cumulative egg production (± SEM) measured at weekly intervals (T1, T2, T3, T4) (*n *= 3). The total reproductive output, quantified as the AUC, was greater for the artificial diet group. This difference was assessed using Welch’s unpaired *t*-test on the AUC values (*t* = 3.53; df = 2.22; *P *= 0.06).

## Discussion

High-quality artificial diets are critical to the industrial development of resource insects during large-scale rearing (Shimizu, 2024). In this study, we characterized the nutritional digestion and absorption traits of the insect by analyzing gene expression profiles of the digestive tract across different developmental stages (using apple‑fed adults and previously obtained larval data). Guided by these transcriptomic traits, we formulated a HP‑1 and showed that it significantly enhances adult growth and fecundity compared to the natural apple diet. Our results demonstrated that high-protein artificial diets outperform the natural diet (apple), effectively enhancing adult fecundity. This finding holds important implications for the advancement of the resource insect industry and global protein supply (Akhtar and Isman 2018, van Huis and Gasco 2023, Kłobukowski et al. 2025).

Transcriptomic analysis revealed significant differences in gene expression between the intestines of adult and larval *P. brevitarsis*, with a substantial number of DEGs identified. The large number of DEGs is biologically expected for a holometabolous insect undergoing complete metamorphosis, reflecting a major physiological and nutritional reprogramming between life stages (Ozerova and Gelfand 2022, Hwang et al. 2023). These DEGs identified in this study were primarily enriched in pathways related to nutrient digestion and absorption, reflecting the adaptability of nutritional requirements specific to different developmental stages. As the core digestive and absorptive organ, the larval midgut exhibited the highest number of annotated expressed genes and unique high-expression genes. It also showed the highest expression levels in 3 core metabolic pathways: carbohydrate digestion and absorption, fat digestion and absorption, and vitamin digestion and absorption, along with the highest number of highly expressed digestive enzyme genes. These characteristics are highly compatible with the nutritional demands of rapid larval growth and development, further confirming its unique ecological advantage in lignocellulosic degradation (Wang et al. 2022). In contrast, although the total number of expressed genes in the adult intestine was relatively lower, the protein digestion and absorption pathway was markedly active, as evidenced by 7 core protease genes (e.g. HCG031453, HCG044724) each exhibiting exceptionally high expression levels exceeding 10,000 FPKM. Additionally, numerous highly expressed digestive enzyme genes related to protein digestion, such as trypsin, serine protease, and aminopeptidase, were identified in the adult intestine. It is important to note that while exceptionally high protease gene expression strongly suggests an enhanced capacity for protein digestion, such expression patterns alone could also reflect non-digestive physiological roles (e.g. in immunity) or specific adaptations to dietary components. Therefore, our inference of a heightened physiological demand for dietary protein in adults is based not only on the transcriptomic profile of apple‑fed adults (high expression of protein digestion genes) but also on the functional validation from feeding trials, where a HP‑1 significantly improved adult growth and reproductive performance. These results indicate that protein is a core nutrient for *P. brevitarsis*, and differences in the protease expression profiles between different developmental stages suggest potential variations in the types of protein nutrition required by different stages. *P. brevitarsis* larvae mainly feed on fermented lignocellulosic plant organic matter (Li et al. 2019), and their protein may be derived from the microbial biomass (Du et al. 2022) produced during fermentation. In contrast, adult *P. brevitarsis* primarily feed on fresh plant tissues such as fruits and milk-stage corn, and their protein nutrition may be more inclined to come from fresh plant tissues (Liu et al. 2025, Zhao et al. 2025). This ecological feeding strategy suggests a possible tradeoff in nature: high-carbohydrate fruits (like apples) provide readily available energy for essential activities such as dispersal and foraging, while protein requirements for reproduction may be met opportunistically from other dietary components (e.g. pollen, microbes) or by selecting protein-rich microhabitats for oviposition. This contrasts with the controlled laboratory setting, where a concentrated and consistent protein supply is necessary to fully realize reproductive potential.

The following feeding experiments using the high‑protein artificial feed further confirmed the practical value of our transcriptomic analysis (performed on apple‑fed adults). The artificial feed had a much higher protein content than apples and was richer in mineral elements (ash). After feeding, the body weight of adults was significantly higher than that of the apple-fed group from the third week onward. It is noteworthy that the slower initial weight gain in the artificial diet group could be influenced by factors beyond gross composition, such as diet palatability, feeding rate, or nutrient assimilation efficiency, rather than nutrient concentration alone (Cohen, 2015). Future studies incorporating precise consumption data will be essential to distinguish these effects. The cumulative egg production of the artificial diet group over 4 wk was 1.62 times that of the apple-fed group, and the protein accumulation in the insect bodies was also significantly increased. These results directly verify the key role of dietary protein in maintaining adult growth and reproductive capacity. In contrast, the higher fat content in the apple-fed group reflects an adaptability to the high carbohydrate content of this natural feed (Shishparenok et al. 2025), aligning with its role in insect energy metabolism.

Collectively, our findings advocate for a stage-specific precision nutrition strategy in mass rearing. This approach leverages the distinct biological roles of each life stage: larvae can be efficiently produced as “bioconverters” on low-value lignocellulosic waste streams, while a separate, nutritionally optimized breeder population (adults) is maintained on a high-protein diet to maximize reproductive output. This framework resolves the apparent contradiction by transforming the larval capacity for waste conversion and the adult requirement for quality protein into complementary pillars of a sustainable production system.

By integrating transcriptome sequencing technology to systematically analyze the gene expression characteristics of the intestines of adult and larval *P. brevitarsis*, combined with feeding experiments and nutritional component analysis of artificial feed and natural feed (apples), this study revealed the molecular mechanisms of nutritional metabolism in different developmental stages of *P. brevitarsis*. It clarified the core nutritional characteristics of “active metabolism of multiple nutrients in larvae and prominent protein demand in adults,” providing a clear direction for the precise formulation of artificial feed. The transcriptome‑guided approach employed here aligns with emerging strategies in insect diet development, as similar methodologies have been successfully applied to optimize artificial diets for other beneficial insects such as *Harmonia axyridis* (Pallas) (Coleoptera: Coccinellidae) and *Arma chinensis* (Fallou) (Hemiptera: Pentatomidae) (Zou et al. 2019, Zhang et al. 2025). Specifically, larval feed should fully utilize their advantage in feeding on fermented lignocellulose, and the ratio of carbohydrates, fats, and proteins in fermentation products should be balanced through fermentation technology control to improve larval growth rate. For adult feed, the priority is to increase the proportion of high-quality proteins. Combined with insect feeding habits, attempts can be made to screen better plant-derived protein raw materials. Although the macronutrient contrast successfully demonstrated proof‑of‑concept, we acknowledge that a follow‑up study using isocaloric, isonitrogenous diets differing only in protein content is required to definitively isolate protein‑specific effects on fecundity. Future refinement of artificial diets for industrial application necessitates comprehensive nutritional profiling—specifically, evaluation of essential amino acid profiles, vitamin and mineral concentrations, and antinutritional factor content—to rigorously optimize protein quality, nutrient bioavailability, and long-term rearing performance. In addition, supplementing with micronutrients that support protease activity (e.g. zinc or other cofactors) may further improve protein utilization efficiency. The economic viability and practical application of such diets at an industrial scale will also require parallel assessment of cost, shelf stability, and manufacturing feasibility. Beyond *P. brevitarsis*, a similar transcriptome‑guided approach could be applied to other economically important insects: first identify life‑stage‑specific digestive enzyme expression patterns, then tailor artificial diets to match the dominant digestive pathways. We also acknowledge that critical parameters such as larval growth rate, development time, survival, and food utilization efficiency were not examined here, as our study focused specifically on adult reproductive performance. Future investigations should include these parameters to fully evaluate the viability of the artificial diet for mass rearing.

## Conclusion

This study identifies a fundamental shift in nutrient requirements between larval and adult *P. brevitarsis*, with adults exhibiting a specific high demand for protein. Guided by transcriptomic evidence of enhanced protease expression, we used a HP‑1 developed by our team. Feeding trials confirmed its efficacy, demonstrating significantly improved adult body weight and enhanced reproductive performance compared to a natural apple diet. These results provide a molecular-to-applied framework for optimizing the mass rearing of this economically important insect.

## Supplementary Material

ieag112_Supplementary_Data

## Data Availability

The raw sequence data reported in this paper have been deposited in the Genome Sequence Archive in the National Genomics Data Center, China National Center for Bioinformation/Beijing Institute of Genomics, Chinese Academy of Sciences (GSA: CRA035722) that are publicly accessible at https://ngdc.cncb.ac.cn/gsa
