# Supplementary material for: Transcriptomic analysis reveals that high-protein artificial diet enhances reproductive performance of Protaetia brevitarsis (Lewis) (Coleoptera: Scarabaeidae)
Source: J Insect Sci. 2026 Oct 5;26(5):ieag112. doi: 10.1093/jisesa/ieag112 (PMC13637846; doi:10.1093/jisesa/ieag112)
Supplement: Supplementary file 1 [file ieag112_supplementary_data.zip › Revised Supplementary_Figures.doc]

**Figure S1 Photograph of the rearing setup for adult *Protaetia brevitarsis*.**

**Figure S2 Principal Component Analysis (PCA) based on the gene expression profiles.** PCA plot was obtained using R (https://www.r-project.org/). pag, adult intestine; plm, larval midgut; plh, larval hindgut.

**Figure S3** Expression level of the differentially expressed genes of *Protaetia brevitarsis* larval midgut (plm) and hindgut (plh).

**Figure S4** KEGG functional annotation of differentially expressed genes between the larval midgut and hindgut of *Protaetia brevitarsis*.
